# Presenting New Ideas and Opinions in Academic Journals

**DOI:** 10.31662/jmaj.2024-0339

**Published:** 2024-12-20

**Authors:** Soichiro Saeki

**Affiliations:** 1 Emergency Medicine and Critical Care, Center Hospital of the National Center for Global Health and Medicine, Tokyo, Japan; 2 Division of Public Health, Department of Social Medicine, Graduate School of Medicine, Osaka University, Osaka, Japan

**Keywords:** Medical Writing, Correspondence, Opinion, Undergraduate, Postgraduate

Writing letters to the editor plays a crucial role in maintaining the integrity of open science, particularly through the critical appraisal of past literature ^(1)^. As previously highlighted by Matsubara ^(2)^, although traditionally a form of commentary, these letters offer authors the opportunity to present new intellectual insights, which can be particularly beneficial for early career researchers ^(3), (4)^ with limited scientific validation. Letters also provide opportunities for those working in clinical practice or with emerging technologies who may face financial constraints.

The author encourages that submitting letters are not only critique existing literature but also present broad opinion pieces. During the COVID-19 pandemic and in the midst of global discussions on racial and ethnic issues, young physicians and medical students shared their perspectives through letters and opinion articles ^(3)^. Although primarily derived from subjective experiences rather than empirical data, these views offer essential insights deserving of academic prominence.

Despite their importance, opinion-based contributions are less common in Japan, especially during the COVID-19 pandemic ^(5)^. The author hopes that this *Journal* will help foster a culture of open discussion in Japan and internationally. Two key factors are necessary to achieve this goal: creating an environment that encourages enthusiasm among the academic community and providing practical guidance to potential authors.

A supportive environment is crucial for promoting writing. The *Journal’*s practice of inviting letters to the editor, as well as responses from authors of referred manuscripts, helps foster dynamic engagement and encourages feedback from readers. This interaction not only validates readers’ voices but also boosts the confidence of letter writers, motivating them to continue contributing to new work. The *Journal’*s inclusion of opinion pieces further promotes valuable discussions, and the author hopes other Japanese journals will create similar environments.

Guidance for potential writers is also essential. Although comprehensive checklists are available for authors writing letters to the editor ^(1)^, this letter covers only Matsubara’s classification ^(2)^ of “Disagreement” and parts of “Agreement” but not “Complimentary” or opinion pieces. Therefore, the author developed a revised version of the previous checklist ^(1)^ for manuscripts offering novel opinions, independent of existing literature (Table 1). This checklist has been made to aid authors in presenting ideas more freely and does not limit the contents to ideas provided in previous articles. This will support authors in crafting well-rounded letters and opinions for academic consideration.

**Table 1.** Checklist for Letters and Opinions.

Sections	Items	✓
Title	Concisely reflects the letter’s main point	□
	Includes relevant keywords	□
Opening	Clearly states the letter’s purpose or objective	□
	Discusses previous references and backgrounds appropriately	□
Body	Presents ideas concisely and clearly	□
	Supports opinion with evidence (references) to maintain scientific soundness	□
	Provides several themes that support the key opinion	□
	Refers to the figure(s)/table(s), if included	□
Closing	Summarizes the key opinion	□
	Provides future perspective if applicable	□

This checklist should be used to write a letter to the editor that mainly focuses on presenting a new opinion, which may or may not reflect a previous manuscript.

The exchange of diverse ideas is critical for fostering sustainable development in research. Broadly disseminating ideas from various sources will drive the future of the medical field.

## Article Information

### Conflicts of Interest

None

### Acknowledgement

The author thanks his colleagues for their helpful discussions on this topic. The author acknowledges the use of Grammarly (Grammarly Inc, San Fransico, USA) for primary language editing. The views expressed in this manuscript are those of the author and do not necessarily represent the author’s institutions.

### Author Contributions

The author is solely responsible for the manuscript content. Artificial intelligence technology was used for the language editing process, and such content was reviewed by the author. The author’s institution played no role in the conceptualization of the manuscript.

### Approval by Institutional Review Board (IRB)

Not applicable.
